# The Physiological Therapeutics of Heart-Failure

**Published:** 1899-12

**Authors:** C. A. F. Lindorme

**Affiliations:** Chicago, Ill.


### THE PHYSIOLOGICAL THERAPEUTICS OF HEART-FAILURE.

By C. A. F. LINDORME, Ph.D., M.D.,

Chicago, Ill.

It is a notorious fact that since some years in the certificates of death heart-failure appears more frequently than ever before as cause.

Without exposing ourselves to a just reproach of presumptuousness, we believe we may give expediency as the cause of this cause; heart-failure is in the pertinent occasion a makeshift ; in the typical cases we refer to, cases like those of President Faure and Bob Ingersoll, heart-failure is so conspicuously the very last of the scenery of life, that it affords the most plausible clue for the dropping of the curtain. But if this makes the position of the attending physician very definite opposite the expectations in the family, it is so indefinite a pathology, that the practitioner may not even derive an inner gratification from his etiology.

As to the resources of materia medica, the physician in the suddenness of the onset is left ; there is hardly time to call in medical assistance, let alone enough to give a trial to remedies, of which nitroglycerine is typical as the *ultima ratio*, and scientific help points altogether to prophylaxis in physiological therapeutics. Broaching this subject, however, methodologically, it seems to us requisite to begin with a scrutiny of physiology, because with a flaw here the therapeutist would be left too, and a flaw in the physiology of the circulation of the blood appears to us by no means entirely out of the question.

The traditional standpoint is in the declaration of William Harvey himself: "Perpetual movement of the blood in a circle is caused by the beats of the heart."—(*John G. Curtis*, in *American Text-book of Physiology.*) But it is owing only to the *vis inertiæ* of a perfunctory conservatism that in this axiom by our great men the merely mechanical side of the display is emphasized, as it was done nearly three hundred years ago by the discoverer of the systematic circulation. Virtually this restriction has been superseded by the later researches. Says W. T. Porter (1 c., p. 368): "The force derived from each contraction (of the ventricles) is generated by the conversion of potential energy, present in the chemical constituents of the muscular tissue, into energy of visible motion, a part also of the potential energy at the same time becoming manifest as heat."

This does not involve a refutation of William Harvey; it is an amplification, a rationalizing, as it were, of the great discovery of the celebrated physician; the merely mechanical fact pointed out by Harvey is given its therapeutical significance by adding the physiological purport. The barren statement of the movement in a circle does not cut by itself any figure, medically. In order to be available for theory and practice we have to know the why and wherefore, and the answer which we have to give to these questions reveals the importance of the latter. There is a hot dispute about the alternative of a muscular or a nervous exhibition of the mechanism of the heart. But it occurs to none of the stalwart champions in the tournament that it is entirely gratuitous, medically, to stand out for the one or the other of the horns of the alternative, so long as the physiological relations of the mechanism are not supplied in the demonstration. It seems to us, even, that in the abovementioned controversy of myologists and neurologists a very ludicrous harmony with regard to a solecism in mechanical doctrine is extant. Either party theorizes a mechanism, *pur et simple*, as the cause of the blood circulation. It is, however, an undeniable fact that *there never was nor ever will be a mechanism which moves or is moved by virtue of its mechanical contrivance.* A mechanism, with reference to its dynamics, depends so entirely and altogether upon the motor-power connected with it, that we may even define a mechanism as *a device to make motor-power available.* At Niagara Falls mechanical contrivances are employed to make the motor-power available which the peculiar bed of the river affords, in its proportion to the gravitation of the water. Now, then, the same mechanism on the plains of Minnesota would not answer the purpose, nor fulfill any theoretical expectations of the kind of the cardiac hydraulics, which they ought to, however, if it was in the mechanism as such that the virtue of the contrivance consisted. William Harvey calls the movement of the blood perpetual, and as a manner to express oneself we can let that pass. But we must not forget that a duplication of the mechanism of the blood circulation, outside the body and minus the chemical constituents which afford the potential energy from which derives the force of the heart's contractions, would be a dead failure, as dead, indeed, as any attempt at a *perpetuum mobile,* by virtue of a mechanical trick only, so far ever has proved. The physiological therapeutics is not likely to derive much benefit from the information, either that it is nervous, or that it is muscular mechanism which we have to assume in the blood-circulation, so long as we do not know what is the motor-power, specifically, which sets the mechanism a going. And in order to make this point, the methodologically correct road, it seems to us, is by way of the question, which, all the urgency of its indication notwithstanding, has experienced a most surprising neglect, the question, *what is it at all that the beating of the heart is for?* There was a time when it was believed that the circulation of the heart, by the continuous movement thereby kept up, prevented the clotting of the blood. But since we know that this deterioration of the vital fluid is precluded by the condition of the coats of the blood-vessels being a faculty of the live tissue, it is only in their physiological relations that we can hope to put clear the enormous biologically mechanical importance of the heart's beats. According to the space devoted by a number of text-books to the cardiac hydraulics, one would suppose that the pumping of the blood was merely for the pumping's sake. But an investigation of all the items of the arrangement gives us the indubitable inference that the only purpose of the blood-circulation, the entire exhaustion of the interest of nature in the mighty and intricate mechanism of the heart's motion, is the *propulsion of oxygen.* Be it ever so indisputable that the blood-circulation serves also the purpose of conveying food to the tissues, our thesis holds for all that. If it was not for the needs of the oxygen the body would require neither heart nor lungs; the capillaries could with the leucocytes and the red corpuscles shift for themselves, just as the lymph fulfills its chemical calling without direct aid by heart or lungs. It is a great biological mistake to suppose that the peculiar animal organisatory complication is necessary to make alimentation, nutrition and assimilation, as also excretion possible, as certainly as the protozoon attends to its physiological cares on its own hook; so it is in the human body only the organisatory complication which makes a heart necessary, lungs, and all the rest of the wonderful animal structure. Above all there is absolutely nothing in all physiology to warrant us in setting down alimentation as the occasion for the unremitting effort of the heart, the unrelenting rhythmical regularity all through life, the extraordinarily uninterrupted pulsation of the blood. There is simply nothing to explain this but the needs of a steady connection of the inner body with the outer air, direct communication, which is indispensable for the minute particles, the aggregation of which is the architectural power on which rests the animal organization, being precluded by the morphological peculiarity of the same. And this fact does not only stand out in indisputable anatomo-physiological prominence, but is fruitful of consequences which are of the utmost importance for physiologieal therapeutics. For if the heart's beats are to no other purpose than the propulsion of oxygen, it is evident that the blood circulation is not properly designated by being called a mechanism, but characterized only scientifically, that means fully in keeping with the relevant facts, by calling it a CHEMISM. As in the quotation from Porter has been pointed out, it is the chemical constituents which supply the muscular power of the heart and gives rise to the irritability in which it is exhibited, and consequently the power which we have to consider as original and ultimate in the matter, is CHEMICAL AFFINITY. Without chemical affinity the mechanism of the blood-path, systemic and pulmonary, is like an electric plant without a dynamo. "Recent observers are united on the necessity of oxygen to the working heart." (*Curtis*, 1 c., p. 4, 81.)

This, in our physiological ratiocination, however, leads us further: If the beats of the heart, although with the ulterior cardiac appendage in themselves a mechanism, are in their ultimate relation a chemism, and if the sole purpose of the cardiac arrangement is in the propulsion of oxygen, it follows, that *the central point of the blood circulation is not in the heart*, be it nervous or muscular, *but in the capillary system.* It is only in passing through these and the minute arteries and veins adjoining that the blood fulfils its essential functions; elsewhere it is in transit merely." (Curtis, 1 c., p. 371.) And this transit is an exceedingly secondary affair; as a mechanical item the arterial branching out and the venous branching in from an extensive portion of the entire volume of the circulatory apparatus. Physiologically, however, they are decidedly secondary; the enormous oxygenation of the blood in the aorta benefits this as little as any of the embranchments on the road towards the capillaries, and conversely the venous poverty of oxygen hurts the venæ cavæ as little as any of the small receptacles of the blood rich in carbonic acid. All the importance of the difference is consummate only in the capillaries, and the physiological interest is limited to the capillary area, the organisatory condition of which is appropriate. The hydraulic theory explains the modalities of the passage of the blood, pulsating in the arteries, through the capillaries and the veins, all in it which has reference to the mechanical part of the conveyance. But it leaves necessarily unexplained the endosmosis and exosmosis in obedience to the biddings of affinity, all, in short, which has reference to the chemical relations, the wonderful life-giving exchange of oxygen and carbonic acid.

"Nutrition (or assimilation), reproduction, conduction, and contractility" . . . form the important features which we may recognize as in all living things, and which we make use of in distinguishing between live and dead matter." (W. H. Howell, 1 c.) Now then does this not point with all cogency of reason to the capilliary system as the source, the biological bed of the organizing power? And is it not rational to consider the heart-mechanism as the outgrowth of this power, instead of assuming the hydraulic mechanism as the moving power in the metabolism of the animal organization? "The collective sectional area of the capillaries is several hundred times that of the root of the aorta." Now if the physiological interest culminated in the inroad into the capillaries by the arterial blood, as merely a liquid pressure-column, and its return to the right auricle with as little loss of pressure as possible, this would be an arrangement of rather questionable wisdom, but it is altogether in accordance with the usual economy of nature, her parsimony in applying the means to her ends, when, in our physiological ratiocination, we avoid the mistake to consider the enormous widening of the blood-path as an integrant part of the mèchanism of the systemic connection of the left ventricle and the right auricle. The most imperious needs is in the conveyance of the oxygen. But since the requisition of the same, because of being unremitting, is exorbitant, nature avails herself of the same mechanism to restitute the loss of oxygen in the blood by returning it in the veins to the aeration-station in the lungs, to keep the heart continuously stocked with the indispensable material. It is moreover a stubborn fact of embryology that the formation of the minute blood-vessels precedes that of the heart, and consequently the purposiveness of the capillary system is established before the heart enters upon its hydraulic service, the coronary arteries sustaining this as much as they presided over the whole formation of the muscle.

The bearing which this change of standpoint has on physiological therapeutics is very considerable. Since the capillary appendage of heart, lungs and blood-vessels is only a substitute of the immediate cell-connection with the cosmical environment, it follows, barring congenital lesions, that, in case of heart-failure, there is invariably presumption of derivative causation: heart-failure as a cause of death is a euphemism; it is death by *cardiac overstrain.* We can tell by the sudden change of the pulse after a full meal, or a "square" one, as the vernacular is, that the circulation is affected. Thus, if all through life by "square" eating the heart is overtaxed, it is unavoidable that consequences supervene which sooner or later may tell in the organ. Anatomically considered, the heart compares favorably with any muscle in the body, gastrocnemius and glutæus not excepted. There is accordingly no probability of any failing before its time, and if it occurs, it can only be by working "overtime." If there is digestive work done for two with only one heart, then the stoutest heart must fail, but not idiocratically but vicariously, as it were, in doing service out of proportion to the needs of the organism to which it belongs.

As a general principle of physiological therapeutics of heart-failure we must infer from the above that direct medication, if it were practically promising even, is theoretically contraindicated. At any rate cardiac stimulants are contraindicated; in such a case the pharmacological whip does it as little as the whip from the harness-maker in the case of an overload to a poor jade. Moderation is what is needed; if there is time left at all, attention to the capillaries is first in order; sitz-baths, cold ablutions, massage, Swedish movements, all to the purpose of easing up the system by accelerating the resorptive action, thereby lessening the overcharge of the heart in propelling the immoderate amount of oxygen which is required for the regular exchange in the disintegration of tissue. As a general prophylaxis, the main counsel of physiological therapeutics is, however, frugality. If there is a notorious reason of heart-failure, as a cause of death, it is certainly high living; in the most pronounced cases the work was excessive which had been given to the heart to do.

